# Reply to Ishikura, H. What Does Soluble C-Type Lectin-like Receptor 2 (sCLEC-2) × D-Dimer/Platelet (PLT) (sCLEC-2 × D-Dimer/PLT) Mean for Coagulation/Fibrinolysis Conditions? Comment on “Yamamoto et al. Super Formula for Diagnosing Disseminated Intravascular Coagulation Using Soluble C-Type Lectin-like Receptor 2. *Diagnostics* 2023, *13*, 2299”

**DOI:** 10.3390/diagnostics14010042

**Published:** 2023-12-25

**Authors:** Hideo Wada, Akitaka Yamamoto, Katsuya Shiraki, Hideto Shimpo

**Affiliations:** 1 Department of General and Laboratory Medicine, Mie Prefectural General Medical Center, Yokkaichi 510-8561, Japan; katsuya-shiraki@mie-gmc.jp; 2 Department of Emergency and Critical Care Center, Mie Prefectural General Medical Center, Yokkaichi 510-8561, Japan; akitaka-yamamoto@mie-gmc.jp; 3 Mie Prefectural General Medical Center, Yokkaichi 510-8561, Japan

We would like to thank Dr. Ishikura for his kind comment [1] on our recent article describing the early diagnosis of disseminated intravascular coagulation (DIC) or pre-DIC using a super formula that includes soluble C-type lectin-like receptor 2 (sCLEC-2), a novel platelet activation biomarker, platelet count (PLT), and D-dimer [2].

We agree with Dr. Ishikura on his proposal of the sCLEC-2/platelet count ratio (called the C2PAC index) to determine sCLEC2 concentrations per platelet unit and consider this to be an index of platelet activation that is a useful predictor of the progression and diagnosis of sepsis-induced DIC (SID) in patients with sepsis [3]. The difference between SID and sepsis-induced coagulopathy (SIC) is unclear [4]. The area under the curve in both super formulaes sCLEC-2 × D-dimer/PLT and C2PAC was high, thus suggesting that both parameters can be useful for the diagnosis of DIC or pre-DIC, SIC, and SID, as reported by Dr. Ishikura. However, the sensitivity and odds ratio of C2PAC was low for DIC or pre-DIC and the diagnostic ability of C2PAC varied among the various underlying diseases. These findings indicate that sCLEC-2 × D-dimer/PLT is more useful than the sCLEC-2/PLT ratio. In addition, C2PAC may be useful in the analysis of monopathy, but the super formula sCLEC-2 × D-dimer/PLT appears to be more useful for the analysis of DIC, which is caused by various underlying diseases.

Additionally, in patients undergoing neurosurgery for high-grade glioma, the C2PAC index is a potential marker for detecting postoperative venous thromboembolism (VTE) [5]. Elevated sCLEC-2 levels have been reported in patients with pulmonary embolism (PE) and atherosclerotic cerebral thrombosis, thrombotic microangiopathy, and coronavirus disease 2019 [6] but not in those with deep vein thrombosis (DVT). In that study, VTE may have included many patients with PE [5], and post-operative effects may cause platelet activation in patients with VTE.

Although Dr. Ishikura suggested that the super formula sCLEC-2 × D-dimer/PLT might be a coagulation and fibrinolysis marker, we consider it to be a biomarker of the activation of coagulation, platelet, and fibrinolysis with the consumption of clotting factors. Indeed, previous reports noted that DIC includes both hypercoagulability and hyperfibrinolysis, which are associated with the marked consumption of clotting factors [7,8]. After the development of sCLEC-2 measurements [9,10], platelet activation has become important for the definition and diagnosis of DIC [2,11]. In addition, most previous diagnostic criteria for DIC have involved complicated scoring systems that require adequate cutoff values for biomarkers and scoring systems [12,13,14]. Biomarkers, especially D-dimer, require standardization [15].

Dr. Ishikura also focused on the importance of the underlying diseases of DIC. The underlying diseases of DIC differed between Ishikura et al. [1,3] and our studies [2,13]. In Dr. Ishikura’s studies, the underlying disease was sepsis in most cases, while the underlying diseases in our study were heterogeneous [2,13]. There are large differences in the sCLEC-2/PLT ratios of patients with sepsis, patients with hematological malignancies, and those with cardiopulmonary arrest (CPA) (Table 1). The sCLEC-2/PLT ratio may indicate platelet activation and consumption, but it does not indicate coagulation and fibrinolysis. Therefore, the C2PAC index may reduce the diagnostic power in the analysis of underlying heterogeneous DIC diseases. In contrast, the super formula sCLEC-2 × D-dimer/PLT includes the activation of coagulation, platelet, and fibrinolysis with the consumption of clotting factors, suggesting that the diagnostic ability for DIC would be retained in the analysis of underlying heterogeneous diseases of DIC. In addition, the super formula sCLEC-2 × D-dimer/PLT does not use a scoring system that requires adequate cutoff values, indicating that it would be simple and easy to apply. As there was a large difference in the super formula sCLEC-2 × D-dimer/PLT between patients with and without DIC, sensitivity and specificity would be high, which suggests that the standardization of D-dimer may not be required for the diagnosis of DIC. In conclusion, the super formula using sCLEC-2 × D-dimer/PLT is more useful for diagnosing DIC or pre-DIC than the sCLEC-2/PLT ratio.

## Figures and Tables

**Table 1:** Receiver operating curve analysis of the super formula for the diagnosis of DIC and pre-DIC using soluble C-type lectin-like receptor 2 (DIC + pre-DIC vs. non-DIC).

**Soluble C-Type Lectin-like Receptor 2/Platelet Count Ratio**						
	**AA**	**CPA**	**INF**	**Neoplasm**	**Trauma**	**All**
Cutoff	18.0	19.2	16.0	14.1	19.3	17.4
Sensitivity	84.7%	86.3%	78.2%	69.5%	86.8%	81.8%
AUC	0.883	0.944	0.892	0.824	0.900	0.895
Odds ratio	16.7	35.7	12.9	4.7	45.3	20.5
**Soluble C-Type Lectin-like Receptor 2 × D-Dimer/Platelet Count**						
	**AA**	**CPA**	**INF**	**Neoplasm**	**Trauma**	**All**
Cutoff	177	153	132	209	163	170
Sensitivity	91.0%	90.0%	87.9%	94.1%	90.3%	89.6%
AUC	0.969	0.944	0.961	0.983	0.982	0.961
Odds ratio	70.5	78.5	50.6	127	65.5	74.6

DIC, disseminated intravascular coagulation; AA, aortic aneurysm; CPA, cardiopulmonary arrest; INF, infection; AUC, area under the curve.
